# Reducing Dietary Sodium and Improving Human Health 2.0

**DOI:** 10.3390/nu15234965

**Published:** 2023-11-30

**Authors:** Pedro Moreira, Carla Gonçalves

**Affiliations:** 1 Faculty of Nutrition and Food Sciences, University of Porto, 4150-180 Porto, Portugal; 2 Epidemiology Research Unit and Laboratory for Integrative and Translational Research in Population Health, Institute of Public Health, University of Porto, 4050-600 Porto, Portugal; carlagoncalves.pt@gmail.com; 3 CIAFEL—Research Centre in Physical Activity, Health and Leisure, Faculty of Sport, University of Porto, 4200-450 Porto, Portugal; 4 CITAB—Centre for the Research and Technology of Agro-Environmental and Biological Sciences, University of Trás-os-Montes and Alto Douro, 5000-801 Vila Real, Portugal

This Special Issue of Nutrients, “Reducing Dietary Sodium and Improving Human Health 2.0”, includes a review article by Jachimowicz-Rogowska and Winiarska-Mieczan [1] that emphasizes the importance of reducing dietary sodium intake to improve public health and provides recommendations for various stakeholders, including food companies, governments, consumers, and healthcare professionals, encouraging them to work together and create synergies to achieve this goal. It also highlights the challenges and opportunities associated with efforts to reduce salt in the food industry. 

Sodium is an essential nutrient which plays crucial roles in various physiological functions, specifically in regulating the volume and systemic distribution of total body water, enabling the cellular uptake of solutes, and generating transmembrane electrochemical potentials through its relations with potassium [2,3]. However, dietary sodium deficiency is rare in healthy populations [2]. In some isolated communities such as the Brazilian Indian Yanomami and Xingu, as well as the Papua New Guinean and Kenyan populations, sodium intakes may be quite low, as reflected by sodium urinary excretion levels ranging from 0.2 to 51.3 mmol/day [4]. At the level of the global adult population, estimates based on the Global Burden of Disease data suggest the opposite extreme, with an average sodium intake of approximately 6 g/day [5], three times higher than the WHO recommendation for an intake of <2 g/day [6], leaving no doubt as to the urgent need for public health interventions aiming to shield populations from exposure to values higher than those outlined in these guidelines. However, the recommended intake levels reflect different interpretations as to what constitutes a high or desirable level of sodium intake, which have been challenged. According to some authors, the recent global estimates of sodium intake may be defined as very “high” [7], while others consider them to be within the “physiologically set normal range” or “moderate” [8]. Therefore, there are many challenges related to sodium data collection, dietary assessment, and the determination of sodium’s impact on health, as well as the expected margins of sodium reduction, considering the roles of different regulators and stakeholders and public health policies [9].

Salt, as the main supplier of sodium in the diet, may come from different sources depending on the studied population. In the case of developed countries, 75–80% of salt intake comes from processed foods, so the focus is often on reducing sodium intake through the nutritional reformulation of food products by the industry seeking to reduce the use of sodium in a gradual and sustained manner, while in developing countries, most salt is acquired from homemade foods [10,11]. However, considering the fact that a country’s per capita gross domestic product [11], food preferences [12], cooking methods [13], and sociocultural contexts [14,15] may all be associated with sodium consumption, understanding these factors and their complex inter-relationships is critical for tackling each of the specific dietary sources of sodium (including those inherent in foods and those added during processing, cooking, or at the table) and designing interventions tailored to specific populations (including those less frequently studied, such as pregnant women, children, or the elderly) and demographic characteristics.

To facilitate the design and implementation of effective salt reduction interventions embracing multi-component approaches and involving governments, the education sector, and the food industry [16], the World Health Organization (WHO) has established global benchmarks for sodium levels across different food categories [17], which are of particular importance for the nutrition reformulation of food products. Recognizing the cost-effectiveness of population salt intake, 194 countries set a goal of reducing their global sodium intake by 30% by 2025 [18]. However, progress has been so slow that this target has been extended to 2030 [19].

If global excessive sodium intake persists, several undesirable consequences for cardiovascular health can be expected, considering that higher amounts of sodium have been consistently linked to a higher risk of hypertension [20] and cardiovascular disease (CVD) [21], and a linear relationship between urinary sodium excretion and hypertension has been described across the entire range of sodium exposure. This evidence supports the validity of recommendations to reduce sodium intake in both normotensive and hypertensive adults in order to prevent CVD [20]. However, there is still controversy in observational studies regarding the types of associations between sodium intake and cardiovascular events and mortality. Some authors [22,23] have used a U- or J-shaped curve to describe this association, while others have shown a linear association between sodium intake and the occurrence of CVD [21,24]. Other authors have argued that rather than looking to the intake of sodium in isolation, one must look at the intake ratio of sodium/potassium, since the intake of potassium can mitigate the detrimental effects of sodium, specifically on CVD [21].

Possible explanations for these different types of associations and the precise levels of sodium that increase the risk of CVD may be related to methodological challenges such as heterogeneity across study populations, the “personal salt index” [25], measurement errors, confounding, reverse causation, or the adverse biological effects observed at low levels of sodium intake [24], namely, the activation of the sympathetic nervous system, serum cholesterol and triglyceride levels, or the activation of the renin–angiotensin system [26], although this has not recognized by all researchers [7].

A higher intake of sodium may lead to other conditions beyond CVD, targeting several organ systems (e.g., the kidney, brain, bone, gastrointestinal tract, immune system, as reviewed by Vinitha et al. [27]), or inducing metabolic changes that may impact the gut microbiome [28], energy balance, and obesity development (reviewed by Wu et al. [29]).

Further research focusing on the various strategies used to address this public health concern, as well as their evaluation on the regional and national levels, is needed, and several research gaps need to be filled [2], including the following areas: controlling the role of energy intake in moderating the effects of sodium intake in disease; the health effects of different molar ratios of sodium/potassium intake; the health effects of early exposure to different levels of sodium, including those in the prenatal environment, the first 8000 days of life, in aging, and in individuals with certain health conditions; the precise characterization of the level of sodium exposure capable of modifying the normal physiological response activating the renin–angiotensin system; the role of sodium intake in the pathogenesis of kidney disease in the general population; the individual characteristics (e.g., genetics) that may lead to different profiles of salty taste perception or to the expression of a particular “personal salt index” [25]; expanding the methods of assessing sodium intake, from new digital health tools (e.g., smartphone apps [30]) to devices and technologies for controlling salt addition in meal preparation at home or in catering [31,32,33]; behavioral change programs and modeling techniques for assessing the potential impact of interventions on salt intake and health outcomes; and the impacts of policy and legislative approaches for reducing salt consumption (e.g., mandatory or voluntary sodium limits on food products, labeling regulations, and taxation).

To summarize, collaboration among researchers, industry stakeholders, communities, and policymakers is essential for addressing these challenges in a comprehensive manner, and significant steps could be taken to improve public health and reduce the burden of sodium-related diseases.
